# Functional Coupling of the Locus Coeruleus Is Linked to Successful Cognitive Control

**DOI:** 10.3390/brainsci12030305

**Published:** 2022-02-24

**Authors:** Marcus Grueschow, Birgit Kleim, Christian Carl Ruff

**Affiliations:** 1 Zurich Center for Neuroeconomics (ZNE), Department of Economics, University of Zurich, 8006 Zurich, Switzerland; christian.ruff@econ.uzh.ch; 2 Department of Experimental Psychopathology and Psychotherapy, University of Zurich, 8050 Zurich, Switzerland; b.kleim@psychologie.uzh.ch; 3 Department of Psychiatry, Psychotherapy and Psychosomatics, University of Zurich, 8032 Zurich, Switzerland

**Keywords:** response conflict, executive function, arousal, functional connectivity, parietal cortex, ventral striatum, dorsal striatum

## Abstract

The locus coeruleus (LC) is a brainstem structure that sends widespread efferent projections throughout the mammalian brain. The LC constitutes the major source of noradrenaline (NE), a modulatory neurotransmitter that is crucial for fundamental brain functions such as arousal, attention, and cognitive control. This role of the LC-NE is traditionally not believed to reflect functional influences on the frontoparietal network or the striatum, but recent advances in chemogenetic manipulations of the rodent brain have challenged this notion. However, demonstrations of LC-NE functional connectivity with these areas in the human brain are surprisingly sparse. Here, we close this gap. Using an established emotional stroop task, we directly compared trials requiring response conflict control with trials that did not require this, but were matched for visual stimulus properties, response modality, and controlled for pupil dilation differences across both trial types. We found that LC-NE functional coupling with the parietal cortex and regions of the striatum is substantially enhanced during trials requiring response conflict control. Crucially, the strength of this functional coupling was directly related to individual reaction time differences incurred by conflict resolution. Our data concur with recent rodent findings and highlight the importance of converging evidence between human and nonhuman neurophysiology to further understand the neural systems supporting adaptive and maladaptive behavior in health and disease.

## 1. Introduction

The locus coeruleus (LC) is a brainstem structure that sends widespread efferent projections to many regions in the mammalian brain [1,2]. The LC is the main source of the neurotransmitter noradrenaline (NE), which is essential for neuromodulation underlying the effects of arousal, attention, and cognitive control [3,4,5,6,7]. Specifically, LC-NE is thought to be important for the type of cognitive control that flexibly adapts cognition and action in line with internal goals or external task demands [8]. An important aspect of this is response conflict resolution, which is critical during decision making and adaptive behavior [9,10]. Response conflict emerges when a prepotent habitual response must be withheld in order to decide for an alternative option that better suits the current behavioral aims or rules [8,11]. There is considerable variability in the capacity to resolve response conflict between individuals, but the neural origins of heterogeneity in this important aspect of adaptive behavior (and its potential links to the LC-NE system) are currently not well understood.

A prominent model of cognitive control postulates that the current level of behavioral conflict must first be registered and monitored [12,13,14,15] before this information is communicated to regions that subsequently implement appropriate adjustments and resolve the conflict through cortical amplification of task relevant information [8,16,17]. It is typically assumed that the preferential processing of relevant information is supported by a distributed network that includes frontal and parietal cortical structures [18,19]. Interestingly, such selective information processing in frontoparietal networks has been proposed to depend on the release of neuromodulators from subcortical areas [7,20,21,22,23], in particular from the LC-NE noradrenergic system [5,7,24,25]. More specifically, previous work has outlined that the activation of the noradrenergic system increases the signal to noise ratio of currently relevant representations and biases the competition for processing resources across multiple levels of the cortical hierarchy [24,25,26]. However, while research on the human noradrenergic system is increasing [27,28,29,30,31,32,33], direct investigations of LC-NE involvement in adaptive behavior, such as cognitive control and selective attention, is scarce [34,35,36,37]. Specifically, the functional connectivity of the LC-NE and its specific behavioral relevance is a particularly understudied area of research in humans [34,36].

Neurophysiological research on rodents and nonhuman primates has identified the LC-NE as the center of the mammalian arousal system [25,38,39,40] and as being important for determining levels of wakefulness [41,42,43]. In contrast, the LC-NE has largely been regarded as unable to modulate activity in attention relevant specific cortical networks [5]. However, recent studies [44] have proposed that the LC-NE neuromodulatory system may be capable of biasing the processing of task relevant information via thalamocortical and midbrain circuits [5,26]. Specifically, recent advances in optogenetic and chemogenetic manipulation of the rodent brain have revealed novel insights into this function and the connectivity of the LC-NE arousal system [44,45]. For instance, chemogenetically activating the rodent LC-NE leads to rapid reconfigurations of resting state connectivity in multiple networks, including the fronto-parietal network as well as a striato-motor network [44]. Moreover, artificially increasing LC-NE firing in this study also revealed strong noradrenergic innervation and increased NE turnover in the dorsal striatum (caudate nucleus), even though this region is widely thought to lack any NE projections [25,46,47,48,49]. However, it has to be noted that early neurophysiological studies in mice have revealed that the shell of the ventral striatum (nucleus accumbens) has a high number of LC projections [50,51]. Importantly, while these animal studies reveal crucial insights into the connectivity architecture of the LC-NE neuromulatory system, they are largely conducted under some form of anesthesia, leaving it unclear to what degree these connections are behaviorally relevant and whether similar connectivity patterns would also arise in humans. Here, we investigate the functional connectivity of the human LC-NE with these cortical and subcortical structures and investigate their behavioral relevance during an important aspect of adaptive behavior: response conflict resolution.

We focused on response conflict resolution because theoretical work has speculated that it may be closely linked to noradrenergic arousal processes and the functioning of the LC-NE arousal system [6,52,53]. Recent studies have, indeed, provided behavioral and pupillometry (a potential proxy for LC-NE firing [44,54,55,56,57,58,59]) evidence for this link [60,61]. In addition, human neurophysiological data link the LC-NE arousal system to individual differences in conflict resolution [34,35,36,37], but its specific functional contributions are not well understood (see below). Classic cognitive control tasks—such as, for example, the flanker task, the color–word stroop task or the emotional stroop task, which we use here—employ reaction times to quantify the individual’s capacity for conflict resolution [8,12,13]. More specifically, a longer reaction time for conflict trials (requiring conflict resolution) compared to no conflict trials (requiring no conflict resolution) is thought to reflect that conflict resolution incurs processing costs involving detection, monitoring and, eventually, adjusting to the behavioral conflict [8,12]. One dominant account posits that this reaction time increase is smaller for individuals with better capacity to resolve the behavioral conflict, as we have found in a previous analysis of these data [34], that, however, has not investigated the individual relationship of reaction times and behavioral accuracy. Therefore, an alternative account suggests that individuals generally aim to maximize task-performance and employ a speed–accuracy trade-off during conflict resolution, which produces individually increased task-performance with longer reaction times for better conflict resolution [62,63,64]. However, several lines of research have also suggested a role of the LC-NE in response inhibition, implicating that LC-NE activity may relate to longer RTs and reduced impulsivity [65,66,67,68,69]. Here, we test these accounts, by relating individual differences in reaction time induced by conflict resolution to individual differences in choice accuracy. Moreover, we relate these indices to the functional coupling of the LC-NE arousal system, to test the hypothesis derived from animal neurophysiological studies that the LC-NE may enhance its functional coupling with regions of the fronto-parietal network known to be involved in selective attention. In addition we explore whether the connectivity between LC-NE and subcortical areas, such as the dorsal and ventral striatum, previously reported in rodents [44], can also be observed in humans and whether LC-NE connectivity to these areas is related to the efficiency of behavioral conflict resolution.

## 2. Material and Methods

### 2.1. Participants

Forty-eight medical students (*n* = 28 women, mean age = 24 years, *SD* = 1.99) were recruited using standard exclusion criteria (fMRI safety, psychopathology). Written informed consent was provided by each participant. Participation was voluntary. Participants were debriefed and compensated (equal to 35 US$). The Cantonal Ethics Committee of Zurich (KEK) approved all procedures prior to study commencement. The results presented here are an addendum analysis to a prior publication using this data set [34], which reported the functional connectivity of the dorso-medial prefrontal cortex (DMPFC), not the locus coeruleus.

### 2.2. Stimulus Presentation

All stimuli were displayed on a grey projection screen (using the Cogent2000-toolbox, http://www.vislab.ucl.ac.uk/cogent_2000.php, accessed on 11 April 2017, implemented in Matlab, The MathWorks, Inc., Natick, MA, USA) which participants viewed by means of a mirror system mounted atop the MR head coil. The vast majority of participants (46 out of 48) completed two runs (200 trials) of the emotional stroop task (see below), while two participants only conducted one run (100 trials). To optimally spread 100 trials across the 10 min of one functional run time, the intertrial intervals (ITI) for each participant were individually sampled from a gamma distribution using the matlab function gamrnd.m (Matlab, Mathworks) with shape parameter 2 and scale parameter 1 and truncated within 2 and 6 s, yielding a mean ITI of 3.1 s.

### 2.3. Behavioral Task

**Figure 1:**
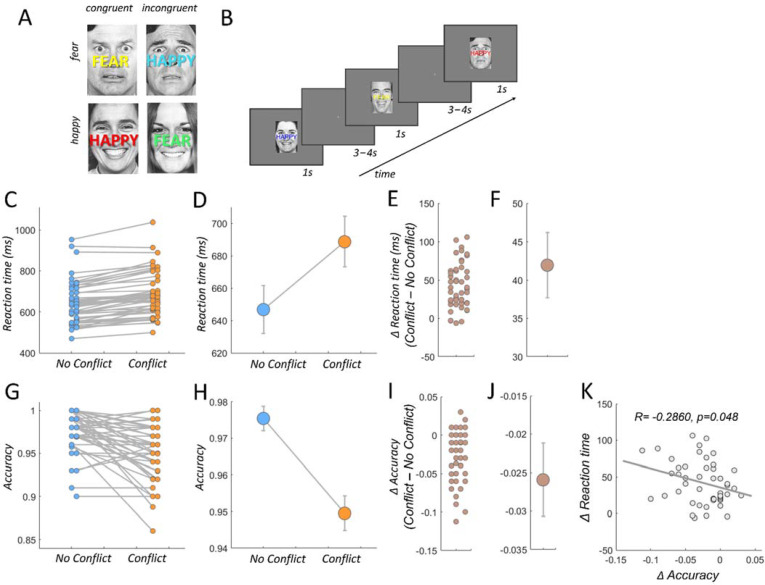
Experimental paradigm and behavioral results. (**A**) The emotional stroop task consists of four possible face/word combinations. Participants identify the emotion of the face and ignore the overlaid word. Adaptation effects were minimized by varying the word color randomly with every trial. (**B**) Trial order presentation example. (**C**) Individual reaction times in congruent (no conflict) and incongruent trials (conflict). (**D**) Mean reaction times in congruent (no conflict) and incongruent trials (conflict). Error bars represent ± 1 standard error of the mean (SEM). (**E**) Individual reaction time differences between no conflict and conflict trials. (**F**) Mean reaction times are significantly increased in conflict versus no conflict trials (*T_47_* = 9.88, *p* = 4.67 × 10^−13^). Error bars represent ± 1 standard error of the mean (SEM). (**G**) Individual response accuracy in no conflict and conflict trials. (**H**) Mean response accuracy in no conflict and conflict trials. Error bars represent ± 1 standard error of the mean (SEM). (**I**) Individual response accuracy differences between no conflict and conflict trials. (**J**) Mean response accuracy is significantly decreased in conflict versus no conflict trials (*T_47_* = −5.25, *p* = 3.65 × 10^−6^). Error bars represent ± 1 standard error of the mean (SEM). (**K**) Individual conflict related reaction time increases are negatively correlated with the individual conflict related decreases in response accuracy (*p* = 0.0488, R = −0.2860).

To induce trials with and without response conflict, we used the emotional stroop task [70,71,72], a wellestablished laboratory procedure involving response conflict [73,74]. The participants task was to categorize faces with respect to their emotional expression (happy vs. fearful). Critically, at the same time, participants had to ignore overlayed emotionally congruent I or incongruent (I) words (“HAPPY”, “FEAR”, Figure 1A,B). The induced processing costs during conflict typically induce higher reaction times (RT) for incongruent than congruent trials [70,71,75] (Figure 1C–F).

The task sequence consisted of fifty no conflict trials (congruent) and fifty conflict trials (incongruent). The varying face/word stimulus combination were shown in pseudorandom order. In addition, the different stimulus categories were counterbalanced for equal numbers of congruent–congruent, congruent–incongruent, incongruent–congruent, and incongruent–incongruent temporal stimulus order (but in the present work we focus on congruency effects only; the trial wise sequence effects along with a more detailed description of the task design are described elsewhere [35]). The participants’ task was to respond as quickly and accurately as possible to the emotionality of the face (press left button for a happy face, press right button for fear or vice versa).

### 2.4. Behavioral Analyses

**Figure 2:**
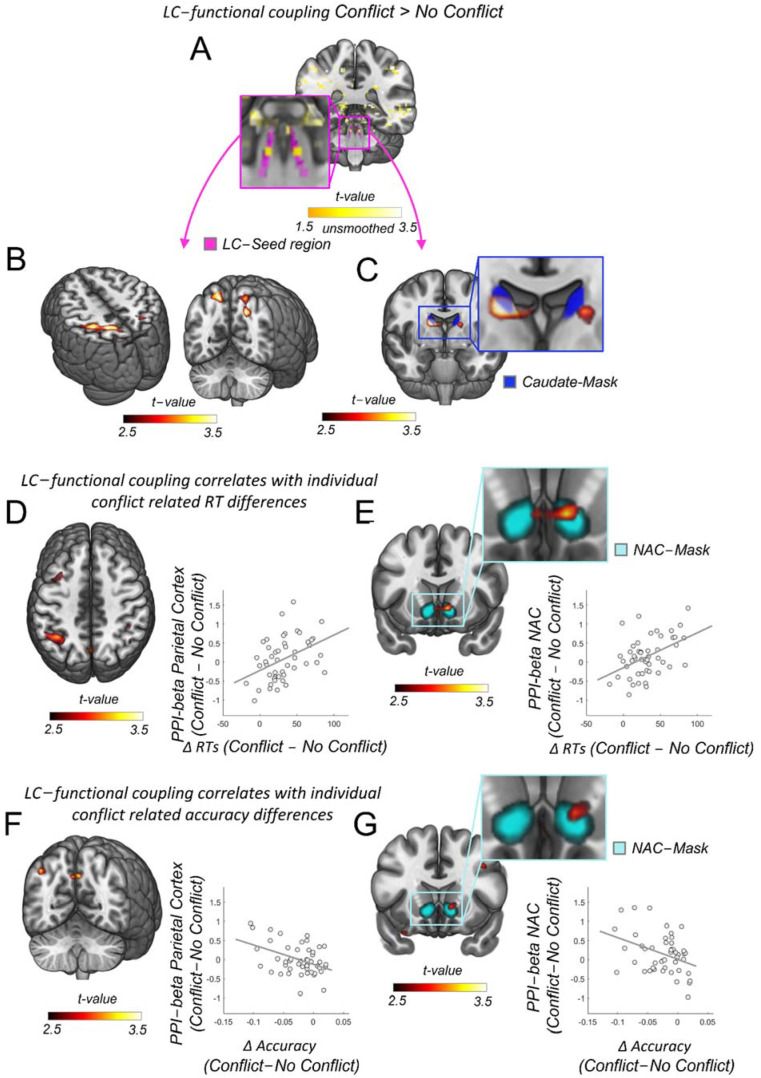
LC functional connectivity to parietal cortex and nucleus accumbens relates to individual differences in reaction times during cognitive control. (**A**) Enhanced activity during conflict versus no conflict trials precisely within the LC-NE 1SD-mask from Keren et al., 2009 (in magenta [77]). Yellow color scale represents the uncorrected average across unsmoothed-physio-corrected I > C individual contrast maps (LC-left: T = 1.77, *p* = 0.41, X/Y/Z: −5/−37/−25, LC-right: T = 1.77, *p* = 0.41, X/Y/Z: −6/−37/−25, N = 48). (**B**) Increased functional coupling between LC-NE and parietal cortex in conflict versus no conflict trials (family wise error corrected), T_(FWE)_ = 5.33, P_(FWE)_ = 0.005, X/Y/Z: −30/−42/48, k = 149). (**C**) Increased functional coupling between LC-NE and the dorsal striatum in conflict versus no conflict trials (small volume peak corrected (SVC) in the bilateral caudate nucleus, T_(SVC)_ = 4.37, P_(SVC)_ = 0.008, 13/−5/18). Caudate nucleus mask in blue. (**D**) Individual increases in LC-NE functional connectivity to the parietal cortex due to conflict correlate with individual conflict-related reaction timeIcrIes (FWE), I_(FWE)_ = 4.14, P_(FWE)_ = 0.018, X/Y/Z: −40/−55/48). For visualization purposes: the correlation between the response conflict resolution score in reaction time and the functional coupling between the LC-NE and the peak voxel in the parietal cortex (voxel coordinates at X/Y/Z: −40/−55/48, N = 48). (**E**) Individual increases in LC-NE functional connectivity to the ventral striatum due to conflict correlate with individual conflict related reaction time increases (small volume peak corrected (SVC) in the bilateral nucleus accumbens, T_(SVC)_ = 3.87, P_(SVC)_ = 0.021, 11/1/−10). For visualization purposes: the correlation between the response conflict resolution score in reaction time and the functional coupling between the LC-NE and the peak voxel in the nucleus accumbens (voxel coordinates at X/Y/Z: 11/1/−10, N = 48). (**F**) Individual increases in LC-NE functional connectivity to the parietal cortex due to conflict correlate with individual conflict related accuracy decreases at trend level (uncorrected, T_(uncorr)_ = 3.16, P_(uncorr)_ = 0.001, X/Y/Z: −35/−75/28). For visualization purposes: the correlation between the accuracy differences and the functional coupling between the LC-NE and the peak voxel in the parietal cortex (voxel coordinates at X/Y/Z: −35/−75/28, N = 48). (**G**) Individual increases in LC-NE functional connectivity to the ventral striatum due to conflict correlate with individual conflict related accuracy decreases at trend level (uncorrected, T_(uncorr)_ = 2.71, P_(uncorr)_ = 0.005, 11/18/−5). For visualization purposes: the correlation between the accuracy differences and the functional coupling between the LC-NE and the peak voxel in the nucleus accumbens (voxel coordinates at X/Y/Z: 11/18/−5, N = 48).

We acquired reaction times and accuracy rate, which is defined as the proportion of trials with correct identification of the emotional valence of the face expression. We excluded trials with RTs exceeding two standard deviations from the mean (across all trials). These trials entered the fMRI model as trials of no interest [70,71,76] (see below). Correlations between behavioral measures and comparison between conflict (I) vs. no conflict triaI (C) were conducted via Pearson correlation coefficient and paired t-tests, respectively, implemented in the statistics toolbox in matlab. The mean reaction time difference between congruent and incongruent trials (Figure 1E) served as an individual score for response conflict resolution and was regressed against individual functional connectivity difference between these trial types (Figure 2D,E).

### 2.5. The fMRI Image Acquisition

Our participants performed two 9.75 min functional imaging sessions during which they performed the emotional stroop task. Per each session, 225 T2* weighted whole brain echo planar images were acquired via a Philips Achieva 3 T whole body scanner (Philips Medical Systems, Best, The Netherlands) and an 8-channel Philips sensitivity encoded (SENSE) head coil (imaging parameters: 2600 ms repetition time (TR); 40 ms (TE); 37 slices (transversal, ascending acquisition); 2.6 mm slice thickness; 2.5 mm × 2.5 mm in plane resolution; 0.65 mm gap; 90° flip angle). Five dummy scans were obtained and discarded before functional image acquisition started to measure at fully equilibrated magnetic field. Additionally, a high resolution T1-weighted 3D fast field echo anatomical scan was acquired for better registration to MNI standard space (sequence parameters: 181 sagittal slices; matrix size: 256 × 256; voxel size: 1 × 1 × 1 mm; TR/TE/TI: 8.3/2.26/181 ms).

### 2.6. The fMRI Image Preprocessing

For image preprocessing and statistical analysis, we employed SPM12 (Wellcome Trust Centre for Neuroimaging). Each functional image was slice–time corrected using the middle slice acquisition time as reference. Participants’ head motion was accounted for using standard SPM12 realignment procedures. Each individual T1-weighted anatomical scan was coregistered to the mean functional image and normalized to the standard T1-MNI template using the “Unified Segment” procedure, as implemented by SPM12 [78]. We normalized the functional images to the MNI standard brain template with the same transformation. Finally, we spatially resampled to 2.5 mm isotropic voxels, and smoothed the resulting images with a Gaussian kernel (FWHM, 6 mm).

### 2.7. The fMRI Data Analysis

First, a general linear model (GLM) was defined containing the main effects, such as conflict and no conflict trials, to serve as a base model with which we control for these main effects, in order to be able to make unconfounded inference on the psychophysiological interaction (PPI) term indicating functional coupling (see below). The base GLM contained two indicator functions at the onset of each of the two trial types (congruent and incongruent). Trials of no interest (see above) were modelled with an additional indicator function [70,71]. For each voxel the BOLD signal was regressed on these conditions with a standard set of hyperparameters modelling MR image autocorrelations with a first order autoregressive model. Head motion was accounted for with additional regressors of no interest constituting six motion parameters which were obtained during the realignment procedure. Moreover, we also accounted for potential confounding effects of eye movements, blinks and pupil size by including these measures in our GLM. Controlling for pupil size in this analysis aimed at counteracting any potential impact that average visual light reflections may have on activity throughout the brain. We employed an MR compatible infrared EyeLink II CL v4.51 eye tracker system (SR Research Ltd., Ottawa, ON, Canada) to sample the eye related information at 500Hz during functional image acquisition. We defined saccades as eye movements extending 0.5 degrees visual angle [79] and eye blinks as periods of signal loss between 80–2000ms [80]; we accounted for these signal losses by linear interpolation [81]. The regressors for saccades and pupil also contained a parametric modulation of eye movement distance and pupil size, respectively.

Imaging the LC-NE in the brainstem is difficult because of its small size and the surrounding ventricles. Low signal to noise ratio in the brainstem is due to breathing and pulsating artifacts [82]. For that reason, we additionally accounted for physiological noise using nuisance regressors that reflected the time-course of signals in the cerebrospinal fluid (CSF) [83]. To this end, we made use of the individual CSF mask that was created during the nonlinear unified segment procedure in SPM12 (see above). For every voxel within this CSF mask, we extracted the BOLD time series and applied a principal component analysis by means of the matlab function pca.m, which is part of the statistics toolbox (MATLAB, The MathWorks, Inc., Natick, MA, USA, version 2017a). The first five principal components were then entered as additional nuisance regressors in the GLM analysis, to account for physiological noise. This procedure has been successfully applied previously and shown to substantially increase signal to noise ratio in the brainstem [35,83]. Statistical inference was drawn via a random effects General Linear Model using the SPM12 framework. We adIed a whole brain FWE-corrected statistical threshold of *p* < 0.05 with an initial cluster-forming voxel level threshold of T = 3.275 (corresponding to uncorrected *p* < 0.001) [84]. For our hypothesis guided ROI analysis investigating LC-NE functional coupling with dorsal and ventral striatum, we employed a standard small volume peak level correction restricted to a caudate nucleus mask provided by the NITRC Atlas of the basal ganglia (https://www.nitrc.org/projects/atag/, accessed on 23 November 2019) and the nucleus accumbens mask provided by the FSL-Harvard-Oxford-atlas (http://neuro.debian.net/pkgs/fsl-harvard-oxford-atlases.html, accessed on 6 November 2013).

### 2.8. Psychophysiological Analysis (PPI)

To investigate the functional coupling of the LC-NE we used a standard PPI approach as implemented in SPM 12, which adds to the base GLM design matrix that includes the main effects of the task the BOLD time series extracted from a 3mm sphere centered on the peak activity identified with the incongruent > congruent contrast within the standard LC-NE mask (1SD mask from Keren et al. [77], Figure 2A). In addition, we added two interaction terms which reflect the interactions of the extracted LC-NE BOLD time course with the C and I regressors. It is important to note that these PPI interaction terms are not confounded with the main effects and interactions with all other experimental variables because they are part of the base GLM design matrix along with physiological, pupil and eye-tracking data (see above) and, thus, accounted for.

The full list of regressors finally included: ‘congruent trials’, ‘incongruent trials’, ‘trials of no interest’, ‘blinks’, ‘saccades’, ‘pupil size’, ‘congruent trials PPI’, ‘incongruent trials PPI’, ‘6 motion regressors’ and ‘5 physiological noise regressors’. To assess the relationship between conflict related functional coupling and behavioral measures, we correlated the size of the functional coupling difference (I > C) with equivalent behavioral differences in reaction time (RT difference between I > C) and accuracy differences between both trial types (I > C) (Figure 1) in a second level analysis. For brain visualizations (Figure 2), we used the freely available software MRIcroGL (https://www.nitrc.org/projects/mricrogl/, accessed on 2 November 2017).

## 3. Results

### 3.1. Behavioral Results

We replicate and confirm previous effects of response conflict resolution for RTs and accuracy [13,70,85]. As expected, responses to trials requiring conflict resolution (incongruent trials) took more time [75]. A comparison between incongruent (conflict) and congruent (no conflict) trials revealed significantly increased reaction times (*T_47_* = 9.88, *p* = 4.67 × 10^−13^, Figure 1C–F) and decreased accuracies (*T_47_* = −5.25, *p* = 3.65 × 10^−6^, Figure 1G–J), and these two behavioral indices of response conflict were negatively correlated (*p* = 0.0488, R = −0.2860, Figure 1K). In other words, the longer an individual takes, on average, to resolve the conflict, the lower the response accuracy during conflict, speaking against a speed accuracy trade-off. In the next analyses, we identified regions with which the LC-NE showed stronger functional coupling during conflict as compared to no conflict trials. In addition, we looked for regions whose functional coupling strength with the LC-NE reflected the individual conflict related enhancement in RTs, indicating that these functional connections are relevant for efficient conflict resolution.

### 3.2. LC Functional Coupling Relates to Individual RTs during Conflict Resolution

The resolution of conflict incurs processing costs due to detection, monitoring, and, eventually, adjusting the behavioral conflict. These processes require preferential processing of task relevant information, which is thought to be supported by the fronto-parietal network [18,19]. In addition, it has been suggested that such selective information processing in the fronto-parietal networks depends on the release of neuromodulators from subcortical areas [7,20,21,22,23] and, especially, the noradrenergic system [5,7,24,25]. Our results are congruent with such an account: We first found that the LC-NE shows enhanced activity during conflict versus no conflict trials. As recommended for brainstem imaging [86], we employed the uncorrected average across unsmoothed and physiological noise corrected individual I > C contrast maps (LC-left: T = 1.77, *p* = 0.41, X/Y/Z: −5/−37/−25, LC-right: T = 1.77, *p* = 0.41, X/Y/Z: −6/−37/−25, N = 48, Figure 2A). Secondly, we find significantly enhanced functional coupling between the LC-NE arousal system and the fronto-parietal brain network during conflict trials as compared to no conflict trials, in particular, for clusters inIe parietaIortex (T_(FWE)_ = 5.33, P_(FWE)_ = 0.005, X/Y/Z: −30/−42/48, Figure 2B). The level of enhanced functional coupling between the LC-NE and the parietal cortex due to conflict resolution was correlated with the individual cIliI related Iincrease (FWE), T_(FWE)_ = 4.14, P_(FWE)_ = 0.018, X/Y/Z: −40/−55/48, Figure 2D), indicating a strong behavioral relevance for these connections during response conflict adjustment. Similar functional connectivity patterns were also found for regions in the dorsal and ventral striatum, even though these regions have been thought to be devoid of any noradrenergic innervations [25,46,47,48,49]. Specifically, LC-NE functional coupling in conflict versus no conflict trials was substantially enhanced in the caudate nucleus in the dorsal striatum (small volume peak corrected (SVC) in the bilateral caudate nucleus, T_(SVC)_ = 4.37, P_(SVC)_ = 0.008, 13/−5/18, Figure 2C), and the level of enhanced functional coupling between the LC-NE and the ventral striatum, particularly the nucleus accumbens, due to conflict resolution was correlated with the individual conflict related RT increase (small volume peak corrected (SVC) in the bilateral nucleus accumbens, T_(SVC)_ = 3.87, P_(SVC)_ = 0.021, 11/1/−10, Figure 2E). As we observed that conflict induced response slowing and accuracy changes were correlated (Figure 1K), we also tested whether LC-NE functional coupling strength would relate to corresponding changes in accuracy. We, indeed, found such correlations in parietal and striatal regions, but only at an uncorrected trend level (Figure 2F,G). Specifically, the LC functional coupling strength in regions of the parietal cortex and the ventral striatum correlated with the accuracy decreases for conflict versus no conflict trials (uncorrected, parietal cortex: T_(uncorr)_ = 3.16, P_(uncorr)_ = 0.001, X/Y/Z: −35/−75/28, ventral striatum, nucleus accumbens: T_(uncorr)_ = 2.71, P_(uncorr)_ = 0.005, 11/18/−5, Figure 2F,G). Thus, our findings suggest a behavioral relevance of LC-NE coupling in response to conflict resolution, but no involvement in speed–accuracy trade-off (for which we should have seen positive correlations with both reaction time and accuracy increases). Nevertheless, these results suggest that functional connections between LC-NE and the parietal cortex and striatum are recruited during response conflict adjustments and play a functional role for such adaptive behavior.

## 4. Discussion

The resolution of response conflict is a crucial aspect in decision-making and a hallmark of adaptive behavior in animals and humans. The capacity for conflict resolution varies widely across individuals, while the neural origins of this heterogeneity remained elusive. Testing hypotheses derived from animal neurophysiology, we showed that behavioral variability in resolving response conflict in an emotional stroop task related to the functional coupling of the LC-NE arousal system with regions in the frontoparietal network as well as striatal regions. Our results reveal that the stronger the individual LC-NE functional coupling during conflict resolution with the parietal cortex and the nucleus accumbens, the longer the individuals take to resolve the conflict, and the less successful they are at it. While our data, thus, do not support a role for human LC-NE arousal system in implementing speed–accuracy trade-offs, they nevertheless reveal LC-NE involvement in response inhibition during cognitive control, which is more taxing in people who are worse at this cognitive function. These findings are not only relevant for future investigations of maladaptive cognitive control behavior, as prevalent in anxiety and depression, but also for disorders involving maladaptive impulse control behavior, such as addiction, obesity, or attention deficit hyperactivity disorder (ADHD) [87].

Several studies in rodents have previously indicated that fundamental behavioral functions such as sustained attention and response inhibition are modulated by the noradrenergic system [65,66,67,68]. For instance, previous studies have shown that increasing NE by blocking its reuptake in the forebrain enhances the animals’ sustained attention and substantially improves response inhibition [65,66,67,68,69]. Interestingly, a recent study in mice showed that LC-NE stimulation increased goal directed attention and decreased impulsivity, while LC-NE suppression heightened distractibility and increased impulsive responding [21]. Moreover, in this study, LC-NE stimulation during the sustained attention task significantly enhanced attentional control, which led to decreasing premature responding, i.e., less impulsivity and, thus, longer reaction times. Similar effects could be observed in a prior study in healthy humans, which highlighted the selective involvement of NE in response inhibition [88]. More specifically, neurochemical inhibition of central noradrenaline reuptake specifically improved response inhibition but had no effect on probabilistic learning, whereas the inhibition of central serotonin reuptake impaired probabilistic learning with no effect on response inhibition [88]. These converging lines of research in animals and humans implicate the noradrenergic system in goal directed behavior and response inhibition, which is well in line with our findings of enhanced LC-NE connectivity during conflict resolution, during which response inhibition is paramount. More specifically, an essential behavioral part in response conflict resolution is to withhold the prepotent conflicting response until the conflict is solved and the alternative action (button press) can be initiated. To make an informed decision, sustained attention has to be employed to focus on task relevant information and filter out task irrelevant stimulus features [19]. This preferential processing of relevant information incurs processing costs, supported by the fronto-parietal network, resulting in increased reaction times [18,19]. Our data show that the LC-NE functional coupling with the parietal cortex during conflict resolution is strongest for individuals who take longer, potentially resembling mice data where enhanced noradrenergic tone decreased impulsivity [65,66,67,68,69]. However, given our correlational approach, we cannot directly speak to the question of whether enhanced functional connectivity is also reflected in enhanced noradrenergic release from the LC-NE. Specifically, the PPI approach cannot logically identify whether any covariance in the residual fMRI timeseries reflects monosynaptic connectivity between two areas, or joint innervation by a third area, or trial-wise endogenous variation in some cognitive state that simultaneously affects both areas. Our PPI results, therefore, need to be interpreted along all these lines, since there is no principled way to rule out either of these effects.

In the influential conflict-monitoring framework of cognitive control, the dorso-medial prefrontal cortex (DMPFC) detects and monitors the level of behavioral conflict [12,13,14,15]. In this model, DMPFC communicates this information to the dorso-lateral prefrontal cortex (DLPFC), which constitutes an important part of the fronto-parietal network. The DLPFC is believed to be responsible for implementing appropriate adjustments via the cortical amplification of task relevant information and, thereby, helping the resolution of the conflict [8,16,17]. We have recently shown that the DMPFC also connects functionally to the LC-NE during conflict resolution [34] in humans. Similar results have been identified in animal tracing studies, which provided evidence for anatomical [89,90,91] and functional connections between the conflict monitoring DMPFC and the LC-NE [92,93,94,95]. This functional connection may serve as a potential source of information about the current level of conflict to the LC-NE and whether the modulation of parietal cortex is still required for response inhibition, while the processes of conflict resolution are still ongoing via sustained attention to task relevant information.

Until recently, it was unclear whether the LC-NE arousal system even innervates the fronto-parietal network [5] or projects to regions in the striatum [25,46,47,48,49]. Our data suggest a functional coupling between these regions also in humans. Novel insights into the connectivity of the LC-NE arousal system have been made specifically through recent advances in optogenetic and chemogenetic manipulation of the rodent brain [44,45]. More specifically, activating the LC-NE in mice via chemogenetic manipulation induced a massive and rapid reconfiguration of the resting state connectome [44]. These changes were observed in multiple networks, including the fronto-parietal but also the striato-motor-network, suggesting previously unknown noradrenergic innervation in these regions [25,46]. These conjectures were corroborated by this previous study, by its also finding an increased turnover of noradrenaline in the dorsal striatum after artificially increasing LC-NE firing [44]. Our data suggest similar functional connections also in humans, as we found LC-NE functional coupling between the parietal-cortex and with the dorsal and ventral striatum during response conflict resolution. Finding these connections during active behavior in humans and also establishing their behavioral relevance is important because most rodent studies are typically conducted under anesthesia, which precludes any inference on behavioral impact [44].

Our functional connectivity results of the LC-NE arousal system during cognitive control are also relevant for a number of psychiatric disorders with impairments of adaptive behavior and potential malfunctions of the noradrenergic system, such as anxiety, depression, addiction and PTSD [96,97,98,99,100,101,102,103,104]. For instance, it has been demonstrated that photostimulation of LC-NE projections to the amygdala caused noradrenaline release, which, in turn, resulted in anxiety like and aversive behavior in mice [99,105]. A recent human imaging study tested these observations using psychophysiological interaction analysis during cognitive control, as we have employed here, and showed that the functional connectivity between LC-NE and amygdala is a crucial predictor for increases in anxiety and depression symptoms after prolonged exposure to stress [35]. This example, and the current data, therefore showcase how hypotheses derived from animal neurophysiology can inform human studies to test the functional and behavioral relevance of the neural arousal circuits [42]. The combination of novel connectivity methods [106,107,108,109] with behavioral tasks that drive the LC-NE [110,111,112,113,114], as well as the use of indirect measures of LC-NE activity such as pupil dilation and heart-rate variability [114,115,116,117], hold great promise to further our understanding of the LC-NE arousal system and its contribution to various psychopathologies [118,119,120].

## 5. Conclusions

In conclusion, we show that the level of functional coupling of the LC-NE arousal system with the parietal cortex and the striatum during cognitive control in humans is directly related to the individual differences in reaction times, supporting a role in response inhibition. These connectivity profiles are remarkable because it has been generally thought that the LC-NE does not innervate the frontoparietal or striatal regions. Our findings may also hold clinical relevance for psychopathologies with cognitive control impairments, such as anxiety, depression, addiction and PTSD.

## Data Availability

Data will be made available upon reasonable request to the corresponding author.
